# ﻿Microcaddisflies (Trichoptera, Hydroptilidae) of the Baja California peninsula, Mexico: checklist and description of two new species

**DOI:** 10.3897/zookeys.1263.148086

**Published:** 2025-12-10

**Authors:** Mauricio Ramírez-Carmona, Rafael Barba-Alvarez, Atilano Contreras-Ramos, Robin E. Thomson

**Affiliations:** 1 Department of Entomology, University of Minnesota, 219 Hodson Hall, 1980 Folwell Avenue, St. Paul, Minnesota, 55108, USA University of Minnesota St. Paul United States of America; 2 Universidad Nacional Autónoma de México, Instituto de Biología, Departamento de Zoología, Colección Nacional de Insectos, Ciudad de México 04510, Mexico Universidad Nacional Autónoma de México Ciudad de México Mexico

**Keywords:** Biodiversity assessment, caddisflies, *
Hydroptila
*, *
Leucotrichia
*, Nearctic, *
Neotrichia
*, new records, *
Ochrotrichia
*, *
Oxyethira
*, taxonomy

## Abstract

Two new species of Hydroptilidae, *Neotrichia baja***sp. nov.** and *Ochrotrichia rivasi***sp. nov.**, are described from the Baja California peninsula, Mexico. New country records for Mexico of four species are provided: *Leucotrichia mutica* Flint, 1991, *Neotrichia kimi* Keth, 2015, *Neotrichia sepulga* Harris, 1991, and *Ochrotrichia lucia* Denning & Blickle, 1972. In this study, 16 Hydroptilidae species were recorded in the peninsula, and nine were new records for the Baja California and Baja California Sur states. This study represents the first microcaddisfly (Hydroptilidae) diversity assessment of the Baja California peninsula.

## ﻿Introduction

The Baja California peninsula, situated in northwestern Mexico, belongs to the Western subregion within the Nearctic region (Escalante et al. 2021). Due to its separation from the continent 12 million years ago, the area exhibits conflicting biogeographic patterns and many endemic species (Morrone 2021). Desert habitats dominate the Baja California peninsula, yet they contain remnant oases systems representing isolated and relictual areas of diversity (González-Abraham et al. 2010; Morrone 2021). Xeric shrublands are the dominant biome in Baja California, with small areas of tropical dry forest and temperate forest (Rzedowski 2006).

Hydroptilidae is the largest and most diverse family of caddisflies with 2665 species worldwide (Thomson 2023). A total of 202 species of microcaddisflies have been recorded from Mexico (Thomson 2023). Despite 26 caddisfly species records from Baja California (Denning 1964; Vanderplank et al. 2016; Razo-González et al. 2023), the microcaddisflies have been poorly documented in the peninsula. Neither Mosely’s (1937) taxonomic checklist of Hydroptilidae in Mexico nor Bueno and Flint’s systematic catalog of the Trichoptera of Mexico (Bueno-Soria and Flint (1978) reported any microcaddisflies in the Baja California peninsula. Blickle (1979) reported several species of microcaddisflies for northern Mexico, but none for the Baja peninsula. Prior to this work, the only record of Hydroptilidae from the Baja peninsula was the species *Hydroptila icona* Mosely, 1937 (Vanderplank et al. 2016). As the caddisfly fauna remains understudied in the area, this study aims to survey and increase knowledge of the microcaddisfly fauna of the peninsula of Baja California.

## ﻿Material and methods

**Figure 1.**
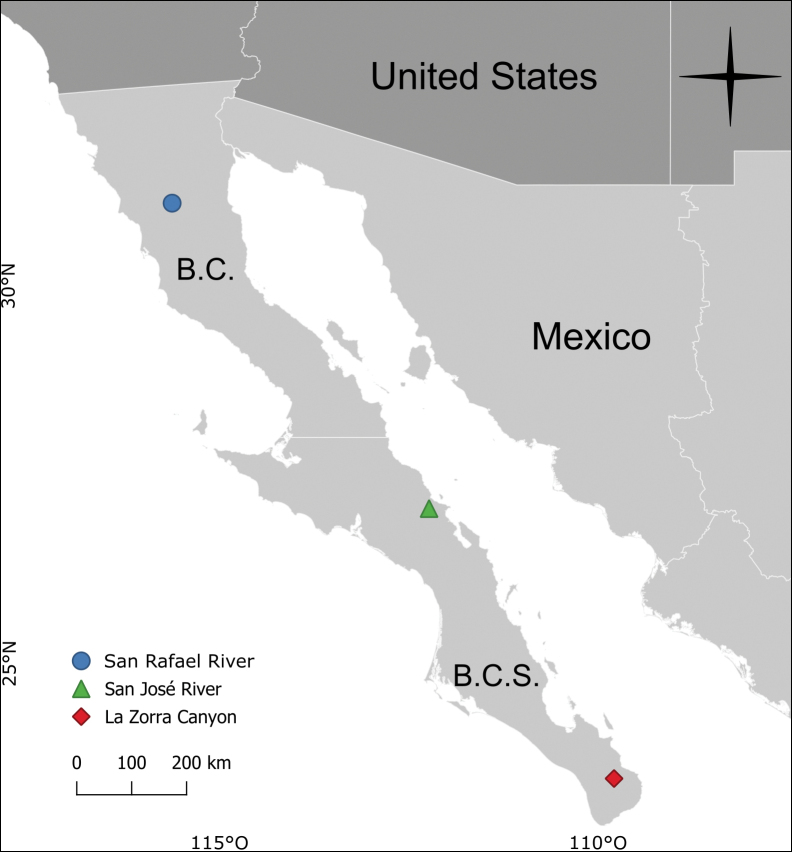
Localities of fieldwork in the Baja California peninsula. B.C. = Baja California; B.C.S. = Baja California Sur.

We collected in three streams along the Baja peninsula during 2021 and 2022 (Fig. 1). Sampling was conducted at night using a white sheet with UV and white lights, using an aspirator; material was either pinned or preserved in 80% ethanol. Specimens were cleared in lactic acid following Blahnik et al. (2007) and examined under a stereo microscope. With a unique alphanumeric sequence starting with the prefix UMSP, each sample was assigned a matrix barcode label. This label is a unique identifier for specimen data uploaded to the University of Minnesota Insect Collection (UMSP) Specify (specifysoftware.org) database. The information for each species is accessible through the Global Biodiversity Information Facility GBIF (gbif.org), which includes distribution maps and latitude, longitude, and elevation. For the descriptions, the specimens were examined at 200x and 400x using a compound microscope with a drawing tube. Final illustrations were completed digitally in Adobe Illustrator (version 29.1). Species descriptions were created using the program DELTA (Dallwitz et al. 1999), following the terminology of Keth et al. (2015) for the genus *Neotrichia* and Marshall (1979) with minor modifications for the genus *Ochrotrichia*. Type specimens and other material examined are deposited in the University of Minnesota Insect Collection, Saint Paul, MN, USA (UMSP), and the Colección Nacional de Insectos, Instituto de Biología, Universidad Nacional Autónoma de México (CNIN). The map was created with QGIS software (version 3.36; Maidenhead; https://qgis.org/).

## ﻿Results

A total of 727 Hydroptilidae specimens were examined, from which 16 species were identified, including two species new to science and four new records for the country (Table 1).

**Table 1.** List of Hydroptilidae species recorded from the peninsula of Baja California. B.C. = Baja California; B.C.S. = Baja California Sur.

Species	Locality	Notes
*Hydroptila arctia* Ross, 1938	San Rafael River, B.C.	New B.C. record
*Hydroptila constricta* Bueno-Soria, 1985	San Rafael River, B.C.	New B.C. record
*Hydroptila icona* Mosely, 1937	San Jose River & La Zorra Canyon, B.C.S.	Reported by Vanderplank et al. (2016)
*Hydroptila rono* Ross, 1941	San Rafael River, B.C.	New B.C. record
*Leucotrichia limpia* Ross, 1944	La Zorra Canyon, B.C.S.	New B.C.S. record
*Leucotrichia mutica* Flint, 1991	La Zorra Canyon, B.C.S.	New country record
*Leucotrichia sarita* Ross, 1944	San Jose River & La Zorra Canyon, B.C.S.	New B.C.S. record
*Neotrichia kimi* Keth, 2015	San Rafael River, B.C.	New country record
*Neotrichia baja* sp. nov.	San Jose River & La Zorra Canyon, B.C.S.	New species
*Neotrichia sepulga* Harris, 1991	San Jose River & La Zorra Canyon, B.C.S.	New country record
*Ochrotrichia lucia* Denning & Blickle, 1972	San Rafael River, B.C. & San Jose River, B.C.S.	New country record
*Ochrotrichia rivasi* sp. nov.	San Rafael River, B.C.	New species
*Ochrotrichia rothi* Denning & Blickle, 1972	San Rafael River, B.C.	New B.C. record
*Ochrotrichia spinulata* Denning & Blickle, 1972	San Rafael River, B.C.	New B.C. record
*Oxyethira arizona* Ross, 1948	La Zorra Canyon, B.C.S.	New B.C.S. record
*Oxyethira parce* (Edwards & Arnold, 1961)	La Zorra Canyon, B.C.S.	New B.C.S. record

### ﻿Taxonomy

#### ﻿ Neotrichia baja

Taxon classificationAnimaliaTrichopteraHydroptilidae

Ramírez-Carmona & Thomson sp. nov.

9DBE04B6-C61E-5F33-8494-0DB6152EECF2

https://zoobank.org/8371249D-55A8-47FA-8E8D-F7448FE02107

Fig. 2

##### Diagnosis.

**Figure 2.**
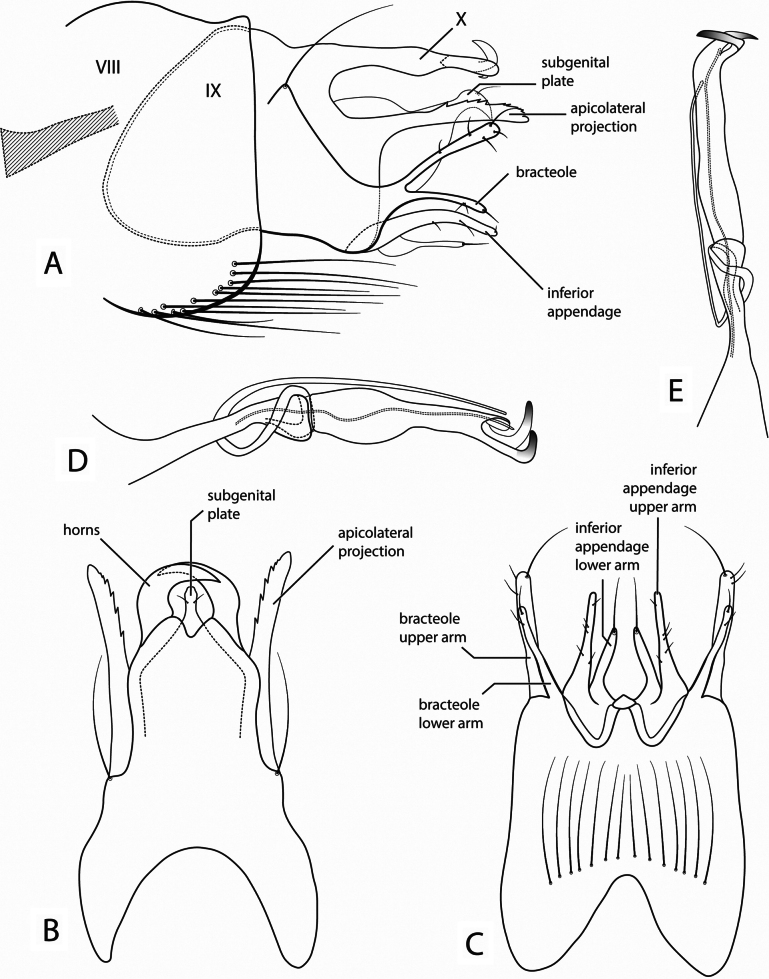
*Neotrichia baja* sp. nov., male genitalia: **A.** Segments VIII–X, lateral (base of phallus cross-hatched); **B.** Segments IX–X, dorsal; **C.** Segment IX, ventral; **D.** Phallus, lateral; **E.** Phallus, dorsal.

This species is placed in the *canixa* group based on the sclerotized horns of segment X, forked bracteoles, and bifid inferior appendages outlined in Keth et al. (2015). This species is most similar to *Neotrichia canixa* (Mosely, 1937). The phallus is very similar, bearing a long paramere, and ending in two sclerotized hooks (Fig. 2D, E). However, the anterolateral margin of segment IX is convex (Fig. 2A), while it is tapered in *N. canixa*; the apicolateral projection is serrate and digitate (Fig. 2A, B), while it is setose in *N. canixa*; and the bracteole is sparsely setose, with only long apical seta in *N. canixa*. Also, the horns in segment X are wider and thicker than those of *N. canixa* (Fig. 2B).

##### Description.

***Male*.** Forewing length 1.7–2.1 mm (*N* = 4). Head unmodified, with 3 ocelli; antennae unmodified. Tibial spur formula 0,2,3. Sternum VII and VIII unmodified, annular. ***Genitalia*.** Segment IX anterior margin convex, in lateral view, with prominent lateral seta, segment fused dorsally with tergum X; apicolateral projection digitate, serrate; bifid bracteole with upper and lower arms slender and digitate, upper arm sparsely setose with prominent apical seta in ventral view, lower arm sparsely setose with prominent apical seta (Fig. 2A). Tergum X extended posteriorly, in dorsal view approximately pentagonal with apical emargination; robust sclerotized horns curving mesally (Fig. 2B). Subgenital plate with hooked apex, dorsally with pair of preapical setae, in lateral view basally membranous (Fig. 2A, B). Inferior appendage bifid, upper arm setose, lower arm with single apical seta (Fig. 2C). Phallus tubular with medial constriction, elongate paramere arising from constriction and extending to apex; apex with pair of slightly sclerotized hooks (Fig. 2D, E).

##### Material examined.

***Holotype***: Mexico • ♂; Baja California Sur: Mulegé, San José de Magdalena River; 27°03.717'N, 112°14.020'W; el. 211 m; 06.vii.2022; A. Contreras, A. Gómez, Y. Marquez, A. Ramírez & M. Ramírez leg.; UV light (in alcohol) (UMSP000281124). ***Paratypes***: Mexico • 2 ♂; Baja California Sur, Los Cabos, Sierra de la Laguna, Rancho Ecológico Sol de Mayo; La Zorra Canyon waterfall; 23°29.829'N, 109°47.592'W; el. 232 m; 14.viii.2021; R. Barba, A. Contreras, M. Luna, Y. Marquez & M. Ramírez leg.; UV light (in alcohol) (CNIN) • 1 ♂ Baja California Sur, Los Cabos, Sierra de la Laguna, Rancho Ecológico Sol de Mayo; La Zorra Canyon waterfall; 23°29.890'N, 109°47.592'W; el. 232 m; 11.vii.2022; A. Contreras, A. Gómez, Y. Marquez, A. Ramírez & M. Ramírez leg.; UV light (in alcohol) (UMSP).

##### Etymology.

Named for the Baja California peninsula, where the specimens were collected.

#### ﻿ Ochrotrichia rivasi

Taxon classificationAnimaliaTrichopteraHydroptilidae

Ramírez-Carmona & Thomson sp. nov.

F70C9448-C6DB-5A33-AE1A-A461ECA5E73E

https://zoobank.org/82962E9C-6EB4-42D2-9317-696FAE013671

Fig. 3

##### Diagnosis.

**Figure 3.**
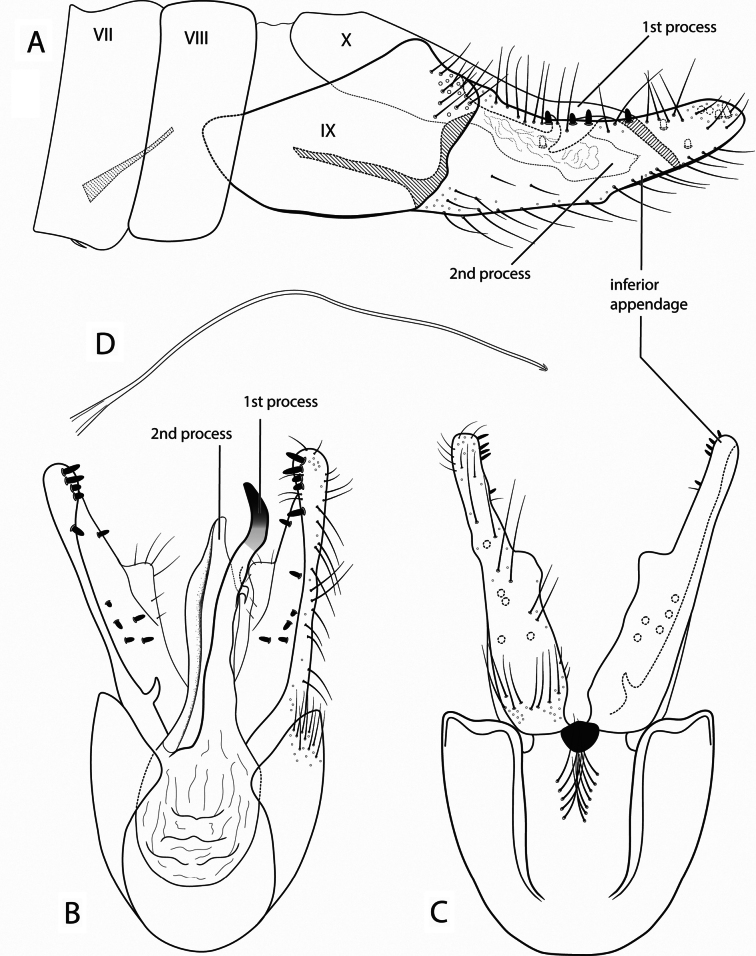
*Ochrotrichia rivasi* sp. nov., male genitalia: **A.** Segments VII–X, lateral (base of phallus cross-hatched); **B.** Segments IX–X, dorsal; **C.** Segment IX, ventral; **D.** Phallus, lateral.

This species is most similar to *Ochrotrichia aldama* (Mosely, 1937). Both have a divided long tenth tergum and an inferior appendage bearing a peg-like setae at the apex. However, the 2^nd^ process of the tenth tergum of *O. rivasi* is not as long and shaped as the 1^st^ process (Fig. 3B); also, peg-like setae are distributed on the apex and along the inferior appendage (Fig. 3A, B), while *O. aldama* only bears peg-like setae at the apex.

##### Description.

***Male*.** Forewing length 3.0 mm (*N* = 3). Head unmodified, with 3 ocelli; antennae unmodified. Tibial spur formula 0,3,4. Sternum VII and VIII unmodified, annular. ***Genitalia*.** Abdomen sternum VII with very short, tapered mesoventral process (Fig. 3A). Segment IX anterolateral margin convex (Fig. 3A), in dorsal view posterior margin fused with tergum X (Fig. 3B). Tergum X membranous basally, asymmetrical with two processes (Fig. 3A); 1^st^ process elongate, with hooked projection near midlength, apex pointed and sclerotized; 2^nd^ process ¾ the length of 1^st^ process, margin irregular, with membranous pocket in lateral view (Fig. 3A, B). Inferior appendage setose, length 3 times greater than width, with slight upward curve, apex rounded; inner surface bearing stout spines; inner surface of right appendage with hook-like basal process (Fig. 3A, B, C). Phallus simple, elongate, threadlike (Fig. 3D).

**Material examined. *Holotype***: Mexico • ♂; Baja California: Ensenada, Rancho Mike´s Sky; San Rafael River waterfall; 31°05.649'N, 115°37.436'W; el. 1232 m; 02.vii.2022; A. Contreras, A. Gómez, Y. Marquez, A. Ramírez & M. Ramírez leg.; UV, White lights (in alcohol) (UMSP000281127). ***Paratypes***: Mexico • 2 ♂; Same data as holotype (in alcohol) (CNIN).

**Etymology**. Named in honor of Dr Gerardo Rivas, an aquatic acarologist at Facultad de Ciencias of Universidad Nacional Autónoma de México, a friend and former advisor of the first author.

### ﻿New country records

The following four species were collected from the Baja peninsula and recorded from Mexico for the first time.

#### ﻿ Leucotrichia mutica

Taxon classificationAnimaliaTrichopteraHydroptilidae

Flint, 1991

C319744F-3C5B-5948-B4CB-ED5090193334

##### Material examined.

Mexico • 3 ♂; Baja California Sur, Los Cabos, Sierra de la Laguna, Rancho Ecológico Sol de Mayo; La Zorra Canyon waterfall; 23°29.890'N, 109°47.592'W; el. 232 m; 1.vii.2022; A. Contreras, A. Gómez, Y. Marquez, A. Ramírez & M. Ramírez leg.; UV light (UMSP).

#### ﻿ Neotrichia kimi

Taxon classificationAnimaliaTrichopteraHydroptilidae

Keth, 2015

152AA794-4715-5F7E-A361-C33A0AA5ADA6

##### Material examined.

Mexico • 10 ♂; Baja California, Ensenada, Rancho Mike’s Sky, San Rafael River waterfall; 31°05.642'N, 115°37.418' W, el. 1224 m. 03.viii.2021; R. Barba, A. Contreras, M. Luna, Y. Marquez, C. Martins & M. Ramírez leg.; UV light (UMSP) • 6 ♂; Baja California: Ensenada, Rancho Mike´s Sky; San Rafael River waterfall; 31°05.649'N, 115° 37.436'W; el. 1232 m; 02.vii.2022; A. Contreras, A. Gómez, Y. Marquez, A. Ramírez & M. Ramírez leg.; UV, White lights (CNIN).

#### ﻿ Neotrichia sepulga

Taxon classificationAnimaliaTrichopteraHydroptilidae

Harris, 1991

FC6DE763-8970-595E-B58F-D261156BEFB3

##### Material examined.

Mexico • 15 ♂; Baja California Sur, Mulegé, San José de Magdalena River; 27°03.731'N, 112°14.066'W, 214 m.; 09.viii.2021; R. Barba, A. Contreras, M. Luna & M. Ramírez leg.; UV light (UMSP) • 3 ♂; Baja California Sur, Los Cabos, Sierra de la Laguna, Rancho Ecológico Sol de Mayo; La Zorra Canyon waterfall; 23°29.829'N, 109°47.592'W; el. 232 m; 14.viii.2021; R. Barba, A. Contreras, M. Luna, Y. Marquez & M. Ramírez leg.; UV light (CNIN) • 12 ♂; Baja California Sur, Mulegé, San José de Magdalena River; 27°03.717'N, 112°14.020'W; el. 211 m; 06.vii.2022; A. Contreras, A. Gómez, Y. Marquez, A. Ramírez & M. Ramírez leg.; UV light (UMSP) • 4 ♂; Baja California Sur, Los Cabos, Sierra de la Laguna, Rancho Ecológico Sol de Mayo; La Zorra Canyon waterfall; 23°29.890'N, 109°47.592'W; el. 232 m; 11.vii.2022; A. Contreras, A. Gómez, Y. Marquez, A. Ramírez & M. Ramírez leg.; UV light (CNIN).

#### ﻿ Ochrotrichia lucia

Taxon classificationAnimaliaTrichopteraHydroptilidae

Denning & Blickle, 1972

90E76DBE-F3D7-5CA2-8707-2F96E498639C

##### Material examined.

Mexico • 28 ♂; Baja California, Ensenada, Rancho Mike’s Sky, trail to waterfall; 31°06.284'N, 115°37.529'W; 1216 m.; 02.viii.2021; R. Barba, A. Contreras, M. Luna, Y. Marquez, C. Martins & M. Ramírez leg.; UV light (UMSP) • 144 ♂; Baja California, Ensenada, Rancho Mike’s Sky, Cascada; 31°05.642'N, 115°37.418' W; el. 1224 m.; 03.viii.2021; R. Barba, A. Contreras, M. Luna, Y. Marquez, C. Martins & M. Ramírez leg.; UV light (CNIN) • 32 ♂; Baja California, Ensenada, Rancho Mike´s Sky; San Rafael River waterfall; 31°05.649'N, 115°37.436'W; el. 1232 m; 02.vii.2022; A. Contreras, A. Gómez, Y. Marquez, A. Ramírez & M. Ramírez leg. UV light (UMSP) • 2 ♂; Baja California Sur, Mulegé, San José de Magdalena River; 27°03.717'N, 112°14.020'W; el. 211 m; 06.vii.2022; A. Contreras, A. Gómez, Y. Marquez, A. Ramírez & M. Ramírez leg.; UV light (UMSP).

## ﻿Discussion

It is remarkable to find 16 species of microcaddisflies in the Baja California peninsula, an area dominated by arid environments. This initial estimate of the richness of the Baja California peninsula highlights a considerable contrast with the fauna observed in other similarly arid areas in the region. When compared to the 40 species of microcaddisflies documented in California, USA (Mendez et al. 2019), there is an overlap of only seven species, all found in the Baja peninsula area (*Hydroptila arctia*, *H. icona*, *H. rono*, *Leucotrichia sarita*, *Neotrichia kimi*, *Ochrotrichia lucia*, and *O. rothi*). In contrast, the Baja California peninsula outnumbers the state of Arizona, USA, which has 15 species of Hydroptilidae recorded (Blinn and Ruiter 2009), presenting an overlap of just four species between both areas (*Hydroptila arctia*, *H. icona*, *H. rono*, and *Leucotrichia limpia*). Overall, the total number of microcaddisfly species observed in the Baja California peninsula currently falls between the total number of each of these adjacent areas.

Studying the fauna of microcaddisflies is important due to their high diversity and the adaptation of larvae to various habitats, yet our knowledge on these species is scarce, as the group has been typically neglected from Trichoptera survey efforts (Thomson 2023). Also, multiple endemic species occur in this predominantly arid area, leaving open the possibility for the discovery of many new hydroptilid species (Morrone 2021). Fieldwork conducted during 2021–2022 revealed five genera: *Hydroptila*, *Leucotrichia*, *Neotrichia*, *Ochrotrichia*, and *Oxyethira*, increasing the known diversity of the family in Mexico to 208 species. These findings contribute to understanding the diversity of Hydroptilidae within this isolated desert landscape, which represents an understudied component of the regional aquatic insect fauna. Although the recorded diversity of Hydroptilidae in Mexico as a whole is above 200 species, studies are needed to address this group’s diversity in this particular unexplored area of the country (Thomson 2023). This study provides the first up-to-date list of Hydroptilidae from the Baja California peninsula and establishes a baseline for future biodiversity and biogeographic studies in the peninsula.

## Supplementary Material

XML Treatment for Neotrichia baja

XML Treatment for Ochrotrichia rivasi

XML Treatment for Leucotrichia mutica

XML Treatment for Neotrichia kimi

XML Treatment for Neotrichia sepulga

XML Treatment for Ochrotrichia lucia

## ﻿Additional information

### Author ORCIDs

Mauricio Ramírez-Carmona https://orcid.org/0009-0006-1216-5947

Rafael Barba-Alvarez https://orcid.org/0000-0003-0049-6862

Atilano Contreras-Ramos https://orcid.org/0000-0001-8044-1348

Robin E. Thomson https://orcid.org/0000-0002-5883-2040

### Data availability

All of the data that support the findings of this study are available in the main text.
